# Once after a full moon: acute type A aortic dissection and lunar phases

**DOI:** 10.1093/icvts/ivab220

**Published:** 2021-08-27

**Authors:** Henrik Bjursten, Daniel Oudin Åström, Shahab Nozohoor, Khalil Ahmad, Mariann Tang, Markus Bjurbom, Emma C Hansson, Anders Jeppsson, Christian Joost Holdflod Møller, Miko Jormalainen, Tatu Juvonen, Ari Mennander, Peter S Olsen, Christian Olsson, Anders Ahlsson, Anna Oudin, Emily Pan, Peter Raivio, Anders Wickbom, Johan Sjögren, Arnar Geirsson, Tomas Gudbjartsson, Igor Zindovic

**Affiliations:** 1 Department of Cardiothoracic Surgery, Lund University, Skåne University Hospital, Lund, Sweden; 2 Division of Occupational and Environmental Medicine, Department of Laboratory Medicine, Lund University, Lund, Sweden; 3 Division of Sustainable Health, Department of Public Health and Clinical Medicine, Umeå University, Umeå, Sweden; 4 Department of Thoracic and Cardiovascular Surgery, Aarhus University Hospital, Skejby, Denmark; 5 Department of Thoracic and Cardiovascular Surgery, Karolinska University Hospital, Stockholm, Sweden; 6 Department of Molecular and Clinical Medicine, Institute of Medicine, Sahlgrenska Academy, University of Gothenburg, Gothenburg, Sweden; 7 Department of Cardiothoracic Surgery, Sahlgrenska University Hospital, Gothenburg, Sweden; 8 Department of Cardiothoracic Surgery, The Heart Centre, Rigshospitalet, Copenhagen University Hospital, Copenhagen, Denmark; 9 Heart and Lung Center, Helsinki University Hospital, Helsinki, Finland; 10 Research Unit of Surgery, Anesthesia, and Critical Care, University of Oulu, Oulu, Finland; 11 Heart Centre, Tampere University Hospital and University of Tampere, Tampere, Finland; 12 Department of Cardiothoracic Surgery, Centre for Cardiac, Vascular, Pulmonary and Infectious Diseases, Rigshospitalet, Copenhagen, Denmark; 13 Department of Surgery, Central Finland Central Hospital, Jyväskylä, Finland; 14 Turku University Hospital, Turku, Finland; 15 Department of Cardiothoracic and Vascular Surgery, Orebro University Hospital and Faculty of Medicine and Health, Orebro University, Orebro, Sweden; 16 Section of Cardiac Surgery, Yale University School of Medicine, New Haven, CT, USA; 17 Department of Cardiothoracic Surgery, Landspitali University Hospital and Faculty of Medicine, University of Iceland, Reykjavik, Iceland

**Keywords:** Dissection of the aorta, Moon

## Abstract

**OBJECTIVES:**

Acute type A aortic dissection (ATAAD) is a rare but severe condition, routinely treated with emergent cardiac surgery. Many surgeons have the notion that patients with ATAAD tend to come in clusters, but no studies have examined these observations. This investigation was undertaken to study the potential association between the lunar cycle and the incidence of ATAAD.

**METHODS:**

We collected information on 2995 patients who underwent ATAAD surgery at centres from the Nordic Consortium for Acute Type A Aortic Dissection collaboration. We cross-referenced the time of surgery with lunar phase using a case-crossover design with 2 different definitions of full moon (>99% illumination and the 7-day full moon period).

**RESULTS:**

The period when the moon was illuminated the most (99% definition) did not show any significant increase in incidence for ATAAD surgery. However, when the full moon period was compared with all other moon phases, it yielded a relative risk of 1.08 [95% confidence interval (CI) 1.00–1.17, *P *=* *0.057] and, compared to waxing moon, only the relative risk was 1.11 (95% CI 1.01–1.23, *P* = 0.027). The peak incidence came 4–6 days after the moon was fully illuminated.

**CONCLUSIONS:**

This study found an overrepresentation of surgery for ATAAD during the full moon phase. The explanation for this is not known, but we speculate that sleep deprivation during full moon leads to a temporary increase in blood pressure, which in turn could trigger rupture of the aortic wall. While this finding is interesting, it needs to be corroborated and the clinical implications are debateable.

**Figure:**
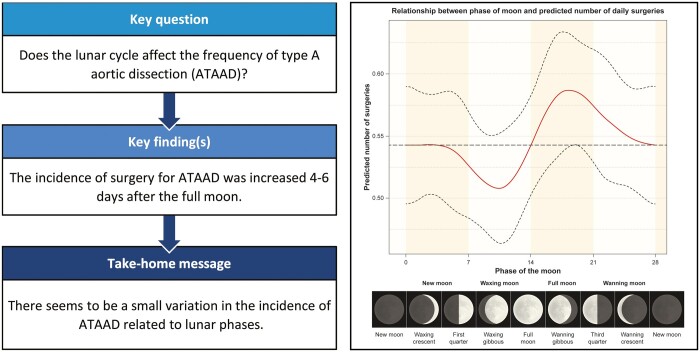

## INTRODUCTION

The impact our nearest celestial body has on our ecosystem, society and humans has been studied and debated for at least 20 000 years [1] when humans first started to record the lunar cycle. Numerous reports have studied both human behaviour and illness in relation to the lunar cycle [2], dividing the public into believers and non-believers, with the latter group receiving widest support from the scientific community [2]. For instance, the notion that birth rate and mental illness increase during full moon has found many advocates and almost become an axiom but has later been dismissed by robust research [2].

However, there is still some available research pointing towards a relationship between the lunar cycle and certain illnesses. Of special interest for the current study are the few reports supporting a relationship between lunar phase and abdominal aortic aneurysm rupture, aneurysmal subarachnoid haemorrhage, and intracranial aneurysm rupture [3–5]. In addition, a recent report found an overrepresentation of acute type A aortic dissection (ATAAD) at full moon [6]. Therefore, one could suspect that there might be an association between the incidence of vascular catastrophes and the lunar phase.

Although several studies have addressed the issue of aortic dissection in relation to the lunar cycle [6, 7], the overall variations of incidence of aortic dissection in relation to the lunar cycle have not been fully established, most likely due to these studies being under-powered. Therefore, the aim of this study was to investigate the relationship between the lunar cycle and the incidence of ATAAD in a large study population based on the Nordic Consortium for Acute Type A Aortic Dissection (NORCAAD) collaboration [8].

## METHODS

### Design

This retrospective, multicentre study included patients (*n* = 2019) who underwent surgery for ATAAD between 1 January 2005 and 31 December 2019 at 8 tertiary centres in Denmark, Finland, Iceland and Sweden constituting the original NORCAAD collaboration [8]. In addition, this study included all patients who underwent ATAAD surgery at Rigshospitalet, Copenhagen, Denmark, and Helsinki University Hospital, Helsinki, Finland, during the same period. In total, 2995 operated patients were included in our analyses, making this one of the largest surgical ATAAD cohorts ever reported. Ethical approval was obtained by each participating centre from Finland and Iceland, whereas national approval was granted for the Swedish (EPN 2019-02097) and Danish centres (STPS 31-1521-67).

### Definitions

The date and time of surgery for ATAAD was used to define the event of aortic dissection. Ideally, the exact time of symptom onset would have been used, but this information was only available for 691 patients in the NORCAAD registry. For those patients where this information was available, the median symptom duration was 7.0 (interquartile range 4.5–13.5) h.

### Full moon definition

The R package *suncalc* was used to define the different phases of the moon as well as to identify the dates when full moon occurred. In the present study, we used 2 different definitions of full moon. In the first definition, the lunar calendar was divided into 4 phases as per the United States Naval Observatory: new moon (days 1–7), waxing moon (days 8–14), full moon (days 15–21) and waning moon (days 22–29) [9]. A binary variable was created to compare full moon exposure to the rest of the phases. In the second definition, full moon was defined as a lunar illumination exceeding 99%. To explore whether the outcome was similar between the 4 specific lunar phases, we compared full moon to the other phases, using full moon as the reference. To further explore differences between the lunar phases, we fitted a generalized additive regression model.

### Statistical methods

The hypothesized association between surgery for ATAAD and exposure to full moon was investigated using a case-crossover design. As each individual serves as its own control, individual time-invariant confounders are adjusted for by design [10]. Control days were selected within the same month and year as the date of surgery and were matched on the day of the week, thus controlling for seasonality, trends over time and day of the week on the outcome pattern [11]. A conditional Poisson regression model with a stratum variable yields identical results to those generated from a conditional logistic regression when there is common exposure across individuals, as was the case in our study where all individuals are assumed to have identical lunar exposure [12].

The methods above present the results for full moon versus the other phases (pooled and separately). To investigate the continuous impact of lunar phase (between 0 and 28 days) and a possible non-linearity of the hypothesized association between phase of the moon and number of surgeries, we used a generalized additive regression model. We assumed that the outcome, daily counts of surgery for ATAAD, followed an over-dispersed Poisson distribution. Our exposure, phase of the lunar cycle, was modelled with a cyclic cubic spline using 6 degrees of freedom across the phases of the lunar cycle.

All results are presented as relative risks (RRs) either as full moon as reference or for full moon exposure, along with their corresponding 95% confidence intervals (CIs). We performed model checks by visual inspection of normally distributed residuals.

In addition, for a subset of the dataset where mortality data were available, we performed non-parametric tests of to analyse the relationship between lunar phases and 30-day and late survival. Intraoperative mortality was assessed using the Kruskal–Wallis test.

R version 3.6.3 was used for statistical modelling.

## RESULTS

A total of 2995 procedures were included in our analyses, of which 789 occurred during full moon according to the first definition (Table 1) and 198 occurred when the lunar illumination exceeded 99% (second definition).

**Table 1:** Distribution of number of surgeries for ATAAD during the different lunar phases and RRs for the outcome of ATAAD surgery according to the first definition with full moon as the reference

Lunar phase	Number of surgeries	RR and 95% CI
Full moon	789	Reference
New moon	743	0.93 (0.84–1.02)
Waxing moon	710	0.90 (0.81–0.99)
Waning moon	753	0.96 (0.87–1.05)

ATAAD: acute type A aortic dissection; CI: confidence interval; RR: relative risk.

**Figure 1:**
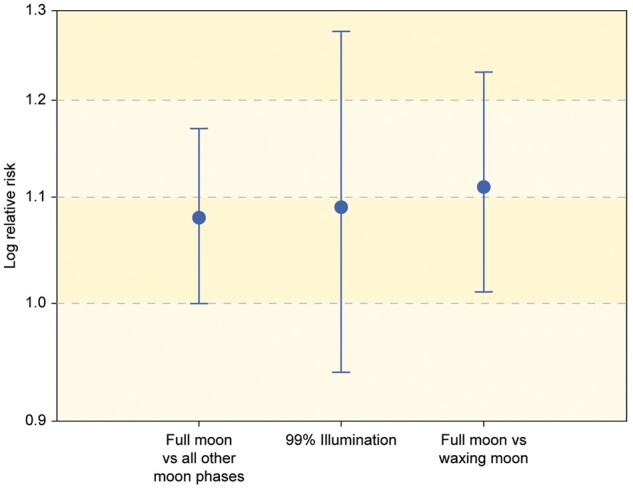
Relative risk with 95% confidence interval for the outcome of acute type A aortic dissection surgery according to the 99% illumination definition and comparison between different moon phases.

We observed an increased risk of surgery for ATAAD on a date corresponding to the first definition of full moon (7 days after full moon) but only to a borderline statistically significant extent (RR 1.08, 95% CI 1.00–1.17; *P *=* *0.057, Fig. 1). There was no significant increase in the risk of surgery for ATAAD using the second (99% illumination) definition (RR 1.09, 95% CI 0.94–1.27; *P *=* *0.255, Fig. 1).

When comparing the 4 phases using full moon as a reference, we observed lower RRs for the other 3 phases. However, only the difference between the waxing moon and the full moon reached the level of significance (RR 0.90, 95% CI 0.81–0.99; *P* = 0.027) (Table 1). When using the waxing moon as reference, the over-risk for ATAAD at the full moon phase was consequently 11% (RR 1.11, 95% CI 1.01–1.23; *P* = 0.027).

**Figure 2:**
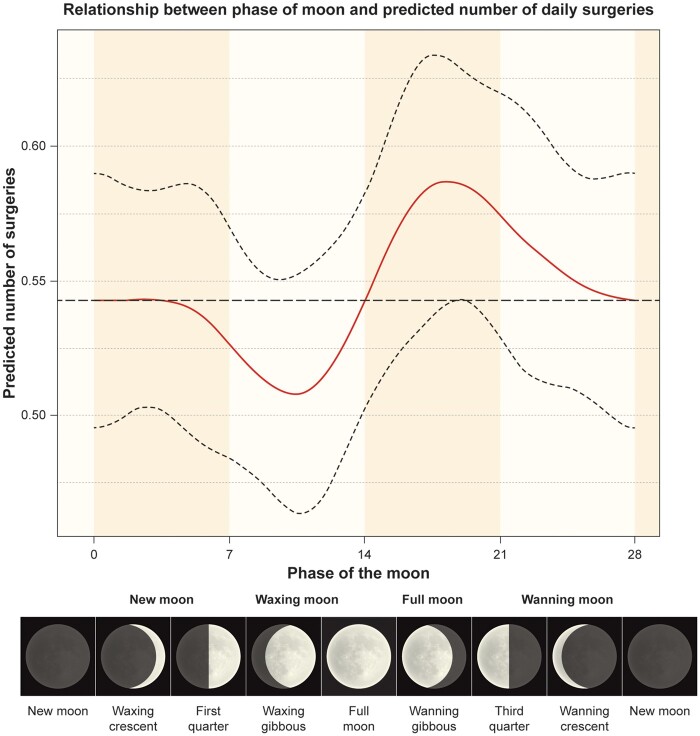
Number of daily surgeries (according to model) for acute type A aortic dissection during different moon phases. Moon phases divided into 4 phases according to the United States Naval Observatory definition [9] directly below graph. The popular name for different illuminations at the bottom of the figure.

The generalized additive regression model regression suggested that the peak incidence of ATAAD did not occur when the moon was maximally illuminated but instead 4–6 days after the actual full moon (Fig. 2).

We observed no differences in 30-day nor long-term survival depending on the moon phase at the date of surgery (log-rank *P* = 0.68 and 0.38, respectively). In addition, we observed no differences in intra-operational mortality between moon phases (Kruskal–Wallis *P* = 0.22).

### Comment

This study demonstrated that the incidence of ATAAD surgery varies throughout the lunar cycle and our data indicate that the incidence peak occurs ∼4–6 days after full moon. When the full moon period was compared to all other phases, the RR was 1.08 and became borderline significant, and when the full moon period was compared to the waxing moon period, the RR was 1.11 with a *P*-value of 0.027. Mortality after surgery did not differ between moon phase for surgery.

Several studies have looked at the relation between lunar phases and the risk of suffering an ATAAD or intracranial Haemorrhage. In a recent study, Ma *et al.* [6] concluded that during full moon, a larger proportion of acute aortic dissections are type A (as opposed to type B). The study pooled data from 2 centres, geographically far apart, however, it did not assess whether there was any variation in the incidence of ATAAD during different lunar phases.

Interestingly, Shuhaiber *et al.* [7] reported that patients undergoing ATAAD surgery during full moon had better survival rates compared to those operated during new moon. These patients also had a shorter length of hospital stay. Studies looking at intracranial Haemorrhage and lunar phases have shown conflicting results. At least 3 studies have found a positive correlation between the 2 [3–5], whereas 2 studies have made the opposite conclusion [13, 14]. These studies, however, were made on smaller cohorts, categorized the lunar phases into 4 or 8 different categories and have therefore not offered the same analysis granularity as the present study.

The potential mechanism for an overrepresentation of ATAAD during and after full moon can only be speculated upon. The gravitational pull from the moon is an often-proposed mechanism in publications assessing the relationship between full moon and disease. The gravitational effect of the moon, often referred to as tidal force, is easy to challenge. First of all, the force of the gravitational pull is negligible. Numerous examples have been presented to illustrate the magnitude of the lunar gravitation on the human body. One famous quote is from Professor Abell of the University of California, Los Angeles, who stated that a ‘mosquito sitting on our arm exerts a more powerful gravitational pull on us than the moon does’. More importantly, the tidal force is diurnal and determined by the Earth’s rotation and not by the 27.3-day orbit of the moon. Proponents point to the combined gravity of the sun and moon when they are aligned as the causative factor. This phenomenon is called ‘spring moon’ and also is seen in the new moon phase, and hence, the effect studied should be the same at both phases, which we did not observe. Moreover, the additive gravitation from the sun is marginal during spring moon [15, 16]. Our finding that the peak incidence was 4–6 days after the actual full moon further supports that the gravitational effect is not relevant in this context.

Hypertension is a well-known risk factor for ATAAD and is present in 67–86% of ATAAD patients [17, 18], and an increase in blood pressure may trigger the actual dissection of the aorta. This phenomenon has been observed in the setting of strenuous physical activity where blood pressure increase is believed to the cause of an ATAAD [19, 20]. Hirst *et al.* [21], in his pivotal 1958 study, reported that a majority of ATAADs occurred during physical exertion. Surprisingly, few publications have addressed blood pressure variation during the lunar cycle directly, but 2 studies report a variation in blood pressure during the lunar phases [22, 23].

In the present study, we observed a peak ATAAD incidence 4–6 days after full moon, and with this in mind, we would like to suggest a third potential mechanism, i.e. sleep variation during lunar phases. Cajochen *et al.* [24] performed a robust study showing that deep sleep was reduced by 30%, time to fall asleep was 5 min longer and total sleep time was reduced by 20 min during full moon. This finding is often used to explain the increase in epileptic seizures during the period after full moon [25]. More important in the context of this study, Lusardi *et al.* [26] showed that sleep deprivation leads to a 5- to 15-mmHg increase in systolic blood pressure and a 35% increase in urine levels of norepinephrine in hypertensive patients. Therefore, sleep deprivation over a couple of days leading to a temporary increase in blood pressure may be the straw that breaks the camel’s back for patients with a locus minori in the ascending aorta. This also explains the findings of Ma *et al.* [6], who found an overrepresentation of ATAAD during full moon, especially given that Landenhed *et al.* [17] showed that hypertension is a major risk factor for ATAAD (54%) indicating that ascending aortic disease is primarily caused by hypertension whereas abdominal aortic disease was driven by smoking and atherosclerosis.

There does not seem to be any association between lunar phases and other cardiovascular events. No studies have been able to detect any correlation between the lunar cycle and acute myocardial infarction, sudden death and acute coronary symptoms [27]. A recent report by Ruuskanen *et al.* [28] looked specifically at stroke and lunar phases in a cohort of 95 000 patients and found no relation. However, a temporary increase in blood pressure does not normally trigger any of these events.

###  

The present study has several limitations. First, only cases that were offered surgery or survived to the point of surgery were included in the database. It is well known that many patients succumb before treatment and that a few patients are turned down for surgery [29]. However, it seems unlikely that the lunar phase would have any effect on those particular mortalities or that the indication for surgery would vary depending on the lunar phase. Second, the database includes only full registration at the time of surgery not the time of dissection. Although there are a few cases of delayed treatment, ATAAD is generally considered a hyper-acute condition requiring immediate surgery. In a subset of patients, we measured time from onset of symptoms to start of surgery, and the median time was 7.0 h, indicating that the delay should have limited effect on our analyses. The study also has a few strengths. First, it encompasses close to 3000 cases of geographically and culturally homogeneous ATAAD patients. We also used a case-crossover design that adjusts for individual level factors (e.g. smoking and body mass index), which makes the cases their own control, and therefore controls for many potential confounders. However, in this type of analysis, there are still many factors that we cannot take into account. However, despite our choice of method, we cannot rule out that residual confounders may still be present in our study.

The present study was able to find an increased incidence of surgery for ATAAD 4–6 days after full moon. Despite the extreme severity of ATAAD, the clinical relevance of this finding is limited as ATAAD is an uncommon condition (3–12 patients/year/100 000 inhabitants) [30], and we observed an 8–11% increase in incidence in the period after full moon. Also, the authors cannot envision any reasonable preventive measures that can be taken.

## ABBREVIATIONS

ATAAD: Acute type A aortic dissection
CI: Confidence interval
NORCAAD: Nordic Consortium for Acute Type A Aortic Dissection
RR: Relative risk
